# Sirt6 regulates postnatal growth plate differentiation and proliferation via Ihh signaling

**DOI:** 10.1038/srep03022

**Published:** 2013-10-23

**Authors:** Jinying Piao, Kunikazu Tsuji, Hiroki Ochi, Munetaka Iwata, Daisuke Koga, Atsushi Okawa, Sadao Morita, Shu Takeda, Yoshinori Asou

**Affiliations:** 1 Department of Rehabilitation Medicine, Graduate School, Tokyo Medical and Dental University 1-5-45 Yushima Bunkyo-ku Tokyo Japan, 113-8519; 2 Department of Joint Surgery and Sports Medicine Section of Cartilage Regeneration, Tokyo Medical and Dental University, Tokyo, Japan 1-5-45 Yushima Bunkyo-ku Tokyo Japan, 113-8519; 3 Laboratory of Veterinary Microbiology, Nippon Veterinary and Life Science University, 1-7-1 Sakaiminamicho Musashino-shi Tokyo Japan, 180-8602; 4 Division of Veterinary Surgery, School of Veterinary Medicine, Nippon Veterinary and Life Science University, 1-7-1 Sakaiminamicho Musashino-shi Tokyo Japan, 180-8602; 5 Department of Orthopedics Surgery, Tokyo Medical and Dental University 1-5-45 Yushima Bunkyo-ku Tokyo Japan, 113-8519; 6 Department of Physiology and Cell Biology, Tokyo Medical and Dental University 1-5-45 Yushima Bunkyo-ku Tokyo Japan, 113-8519

## Abstract

Sirtuin 6 (Sirt6) is a mammalian homologue of NAD^+^-dependent histone deacetylase Sir2. Although Sirt6−/− mice exhibit growth retardation, the role of Sirt6 in cartilage metabolism is unclear. The aim of this study was to investigate the Sirt6 signaling pathway in cartilage metabolism. Immunohistological evaluation of the tibial growth plate in Sirt6−/− mice exhibited impaired proliferation and differentiation of chondrocytes, reduced expression of Indian hedgehog (Ihh), and a senescent phenotype. When Sirt6 was knocked down in chondrocytes in vitro, expression of Ihh and its downstream genes were reduced. Impaired differentiation by Sirt6 silencing was completely rescued by administration of a Hh signal agonist. When sirtuins were activated, chondrocyte differentiation was enhanced together with activation of Ihh signal, and these effects were abrogated by Sirt6 silencing. ChIP assay revealed the affinity of ATF4 to the Ihh promoter was markedly decreased by Sirt6 knockdown. These data indicate Sirt6 directly controls proliferation and differentiation of chondrocytes.

The stress-response and chromatin-silencing factor Sir2, a yeast sirtuin, is a NAD^+^-dependent histone deacetylase and is involved in various nuclear actions^1^. Among the seven mammalian sirtuin family members, sirtuin 6 (Sirt6) is localized to the nucleus and is involved in transcriptional silencing, genome stability, and longevity^2^,^3^. Sirt6 was originally identified as an ADP-ribosyltransferase^4^. Recent studies have demonstrated that the Sirt6 protein is a NAD+-dependent histone 3 deacetylase that targets acetyl-H3K9 and acetyl-H3K56^5^,^6^. Sirt6 deacetylates histone H3 lysine 9 (H3K9) on telomeres and plays a role in their stability^5^. Human Sirt6 also deacetylates C-terminal binding protein interacting protein (CtIP) and promotes DNA end resection^7^. Recent studies have revealed multiple functions of Sirt6 in the regulation of inflammation and metabolism. Sirt6 inhibits inflammation by suppressing NF-κB target molecules via interaction with the RelA subunit of NF-κB and by deacetylating H3K9 at target promoters^8^. In glycometabolism, Sirt6 functions as a corepressor of the transcription factor Hif1α and inhibits glycolysis through suppression of Hif1α activity^9^. As a result, Sirt6 null mice die before 4 weeks of age due to lethal hypoglycemia^4^.

Sirt6−/− mice develop normally for the first two weeks except for reduced body size, which is apparent early after birth^4^. Sirt6−/− mice also exhibit features of premature aging, such as osteopenia and lordokyphosis^4^. Serum insulin-like growth factor 1 (IGF-1) and glucose levels are both markedly reduced in Sirt6^−/−^ mice by the age of 24 days^4^. A recent study demonstrated that neural-specific deletion of Sirt6 in mice leads to postnatal growth retardation due to somatotropic attenuation through low growth hormone (GH) and IGF-1 levels^10^. Thus, Sirt6 contributes to skeletal growth in part through the regulation of IGF-1 secretion. However, the local functions of Sirt6 in postnatal growth of the skeleton are still unknown.

Longitudinal bone growth is organized by growth plate chondrocytes^11^. The growth plate is composed of several zones of chondrocytes including the resting zone, proliferating zone, prehypertrophic zone, and hypertrophic zone^12^. The rate of longitudinal bone growth is higher in early life. Over time, the growth rate declines due to a decrease in the number of cells in the proliferating zone and in the number and volume of hypertrophic cells^13^. When growth plate allografts are transplanted into young or old recipients, the growth rate of the transplanted growth plate depends on the age of the donor animal and not the age of the recipient^14^. This finding indicates that this programmed senescence of the growth plate is due to local mechanisms^14^. Several local factors regulate the process of chondrocyte proliferation and differentiation. Among them, Indian hedgehog (Ihh), a member of the hedgehog family of secreted signaling molecules, plays a central role in normal skeletal development^15^,^16^. Ihh controls the transcriptional activity of Gli proteins through binding to its receptor patched 1 (Ptch1) and through derepression of the signaling receptor smoothened (Smo)^16^,^17^. Ihh regulates chondrocyte proliferation and differentiation via parathyroid hormone–related protein (PTHrP) in a dependent or independent manner^18^,^19^,^20^.

Even though Ihh is one of the key regulators of chondrocyte metabolism, the mechanisms by which Ihh expression is regulated are poorly understood. Runx2 strongly induces Ihh expression in chondrocytes. Moreover, Runx2 directly binds to the promoter region of the Ihh gene and activates the Ihh promoter in vitro^21^. Wnt9a is a temporal and spatial regulator of Ihh. Ihh expression is reduced in embryos double-heterozygous for Wnt9a and β-catenin. The β-catenin/Lef1 complex and the Ihh promoter interact directly^22^. Delta-EF1, a two-handed zinc finger/homeodomain transcriptional repressor, negatively regulates Ihh expression by binding to the putative regulatory elements in intron 1 of Ihh^23^. ATF4 is a leucine zipper-containing transcription factor and a member of the cAMP response element-binding protein (CREB) family. ATF4 plays a critical role in the regulation of chondrocyte proliferation and differentiation during skeletal development via Ihh regulation^24^.

The aim of this study was to investigate the role of the Sirt6 signaling pathway in cartilage metabolism. In this study, we explored the roles of Sirt6 in Ihh-dependent chondrocyte development using Sirt6−/− mice. We identified Sirt6 as a novel regulator gene of Ihh expression in the growth plate.

## Results

**Figure 1:**
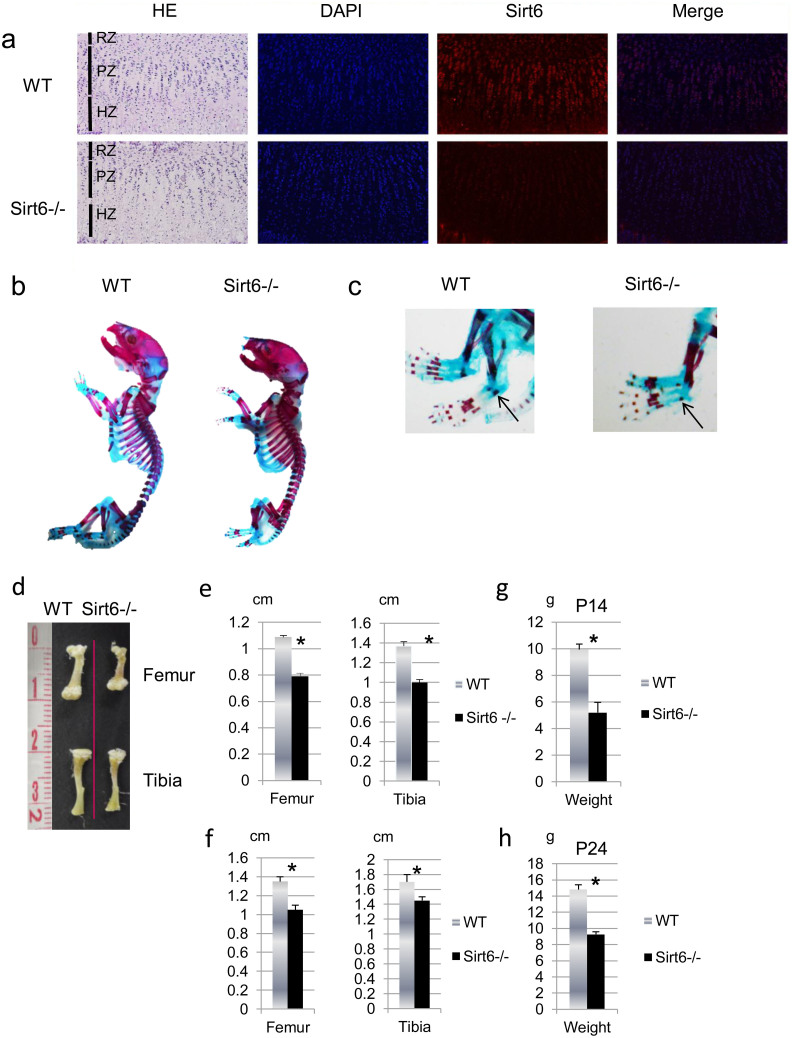
Sirt6 is expressed in chondrocytes. (a) Immunohistological analysis of P14 wild-type mouse growth plates showed Sirt6 is highly expressed at proliferation zone and prehypertrophic zone. Note a lack of immunohistochemical staining for Sirt6 in Sirt6−/− growth plate. RZ: restingzone, PZ: proliferating zone, HZ: hypertrophic zone. (b) Sirt6^−/−^ mice exhibit dwarfism. Alizarin red S- and Alcian blue-stained skeletons of P1 WT and Sirt6^−/−^ mice. Note delayed ossification of metatarsal bone in Sirt6−/− (c, arrows). (d) Quantification of femur and tibia length of P14 WT and Sirt6^−/−^ mice. Values represent the mean ± SD of 4 samples per group. *; p < 0.05.

We first examined the expression of Sirt6 protein in the growth plate chondrocytes by immunohistological analysis. Immunostaining showed Sirt6 protein was highly expressed by the growth plate preferentially in proliferating and prehypertrophic chondrocytes postnatally at 14 days (Figure 1a).

### Sirt6–null mice exhibit dwarfism

Homozygous Sirt6 null mice were found to be smaller than their wild-type (WT) littermates at birth. While there was no abnormal skeletal patterning, delay of ossification of the metatarsal bone in Sirt6−/− mice was detected (Figure 1b, c). The size difference was detectable at birth and continued throughout the growth of the mice. From two weeks to 3.5 weeks after birth, the lengths of the tibiae and femora were 20–30% shorter in Sirt6−/− compared to WT or heterozygotes (Figure 1d–f). The weight of Sirt6−/− mice was also significantly reduced throughout life (Figure 1g, h).

### Sirt6–null mice exhibit defects in proliferation and hypertrophic differentiation

**Figure 2:**
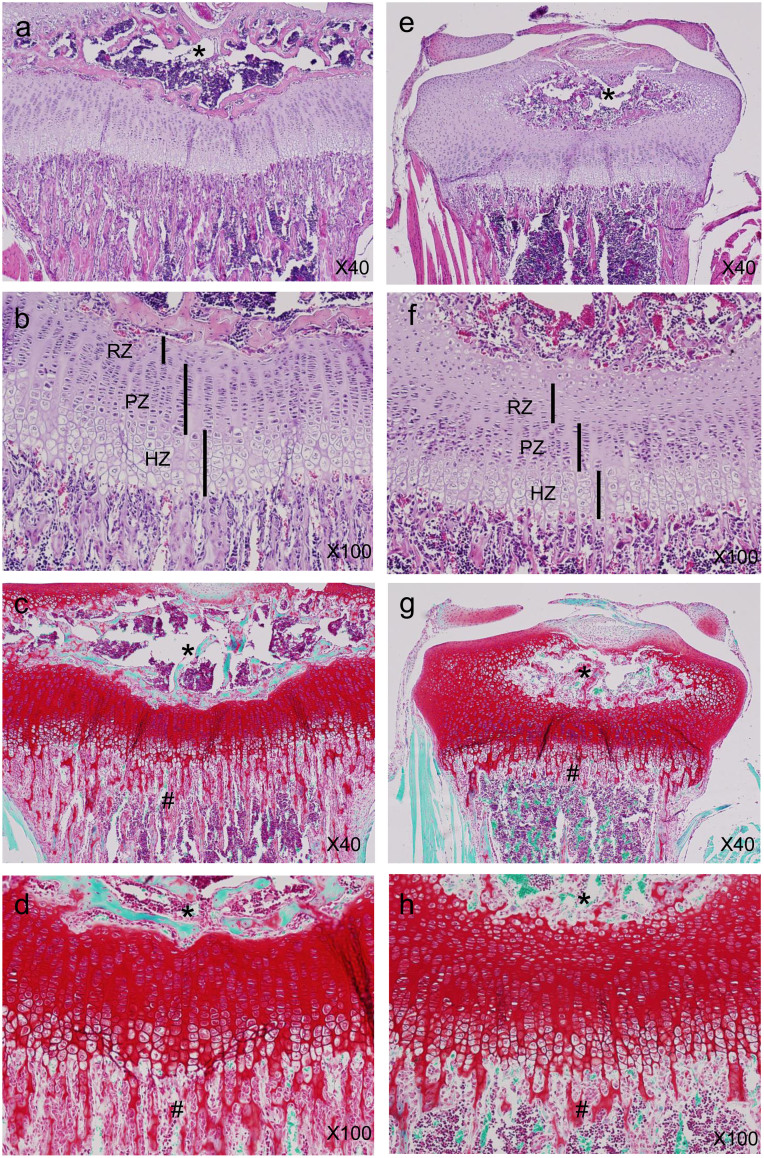
Proximal tibia growth plates from 2-week-old mice were stained with HE (X40 a, e; X100 b, f) and safranin O (X40 c, g; X100 d, h). (a), (b), (e), (f) A reduction in the thickness of the proliferating zone (PZ) and hypertrophic zone (HZ) was observed in the Sirt6−/− growth plates, whereas resting zone (RZ) thickness was increased. (c), (d), (g), (h) Safranin O staining indicated delayed ossification of secondary ossification center (*) in Sirt6−/− mice. Primary spongiosa formation (#) was also markedly reduced in Sirt6−/− mice. Also, proteoglycan expression was sparsely observed in the hypertrophic zone of Sirt6−/− mice (d), (h). Magnification is indicated in the figure.

**Figure 3:**
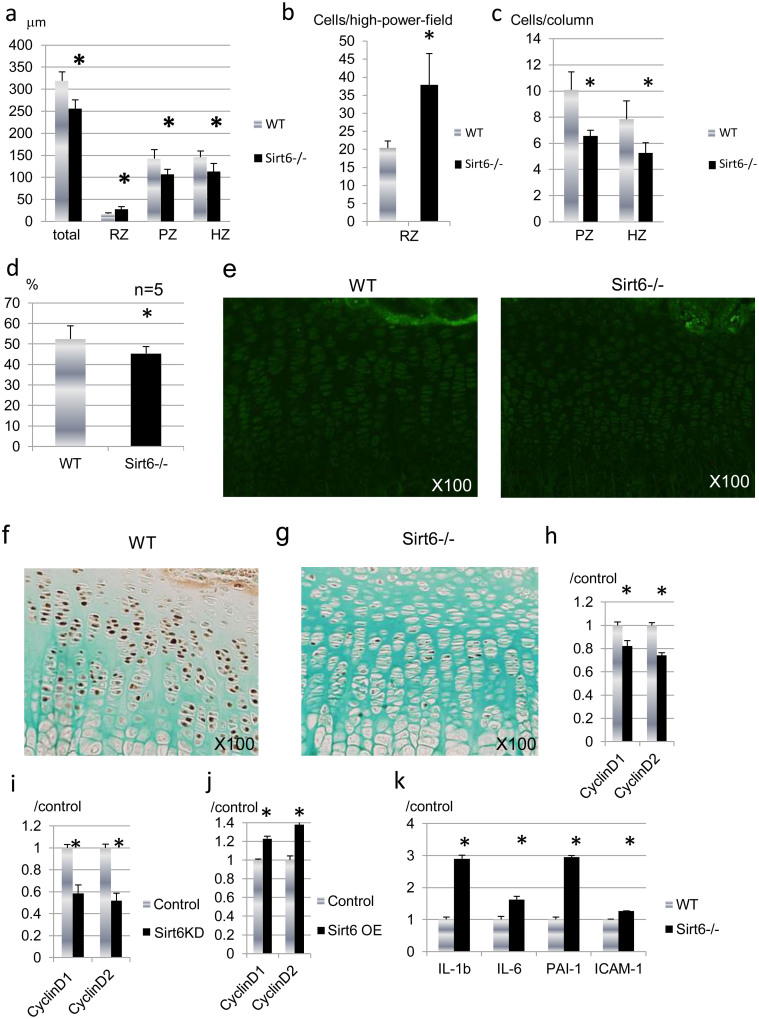
Sirt6 is required for chondrocyte proliferation. (a) Histomorphometric analysis of proximal tibia growth plates from the Sirt6−/− and WT littermate mice confirmed a reduction of approximately 20–30% in the thickness of the proliferating zone and the hypertrophic zone. Also, there were fewer cells per column in the proliferative zone and hypertrophic zone in Sirt6−/− mice (b). (c) The percentage of the hypertrophic zone volume occupied by ECM was reduced in Sirt6−/− growth plates. (d) TUNEL staining of P14 WT and Sirt6−/− mouse femur sections. The number of apoptotic cells was similar between Sirt6−/− and WT. (e), (f) PCNA immunohistochemistry of P14 WT (e) and Sirt6^−/−^ (f) mouse tibia sections. There are fewer PCNA-positive (brown) proliferative chondrocytes in the growth plates of Sirt6^−/−^ P14 pups. (g), (h) Sirt6 siRNA reduced cyclin D1 and cyclin D2 expression in primary epiphyseal chondrocytes (g) and ATDC5 cells (h). (i) Transient overexpression of Sirt6 in ATDC5 cells increased cyclin D1 and cyclin D2 mRNA. Values represent the mean ± SD of 3 samples per group. *; p < 0.05. Magnification is indicated in the figure.

To determine the cause of dwarfism in the Sirt6−/− mice, we compared chondrocyte proliferation and differentiation in tibial growth plates from WT and Sirt6−/− mice at postnatal day 14 (Figure 2). P14 was chosen because this is when serum glucose levels are similar between WT and Sirt6−/− mice. Histological evaluation indicated the resting zone (RZ), which consists of a thin layer of small, undifferentiated cells, was increased in width and cell number in the Sirt6 −/− growth plate compared to WT (Figures 2a, b, e, f and 3a, b).

The proliferating zone (PZ) is composed of columns of rapidly dividing chondrocytes. The length of the PZ and the number of chondrocytes per column were significantly reduced in Sirt6−/− mice (Figures 2a, b, e, f and 3a, c). The thickness of the hypertrophic zone (HZ) and the number of chondrocytes per column were also significantly reduced in Sirt6−/− mice (Figures 2a, b, e, f and 3a, c). As a result, the width of the whole growth plate was significantly reduced in Sirt6−/− mice (Figure 3a). Safranin O staining revealed aberrant staining for proteoglycans, especially in the hypertrophic zone of Sirt6−/− mice (Figure 2d, h). Histomorphometric analysis revealed that matrix-to-cell ratio in the hypertrophic zone was reduced in Sirt6−/− (Figure d), indicating impaired matrix synthesis of chondrocytes by Sirt6 deficiency. Furthermore, the formation of primary spongiosa and ossification of the primary ossification center were apparently impaired in Sirt6−^/−^ mice (Figure 2d, h). The delay of ossification in Sirt6−/− mice continued until P24 (supplementary figure S1), immediately before the end of the life span of Sirt6−/− mice.

To address proliferative and apoptotic activity in the growth plate chondocytes, we performed immunostaining for PCNA and TUNEL staining using sections of tibia. A marked reduction in the percentage of PCNA-positive nuclei (brown) was observed in the proliferative zone in Sirt6−/− mice (Figure 3f, g), whereas the number of TUNEL positive cells was not affected by Sirt6 deficiency at P14 (Figure 3e) and at P1 (supplementary figure S2a). The expression of activated caspase-3, a marker of apoptosis, was also similar between Sirt6−/− and WT (supplementary figure S2b). Thus, Sirt6 signaling is required to maintain the high rate of chondrocyte proliferation observed in rapidly growing long bones in mouse skeletal development.

To determine whether Sirt6 regulates mitogenic responses in chondrocytes, we examined the effect of Sirt6 depletion on the expression of cyclin D1 and D2 in vitro. Depleting Sirt6 reduced cyclin D1 and D2 expression in both primary epiphyseal chondrocytes (Figure 3h) and ATDC5 cells (Figure 3i). In contrast, the expression of cyclin D1 and D2 was enhanced by overexpression of Sirt6 in ATDC5 cells (Figure 3j). These results suggest that Sirt6 promotes cyclin D1 and D2 expression, contributing to the continuation of growth plate chondrocytes in a proliferative state. These data indicate that the decrease in growth plate thickness is caused by reduction in the proliferative ability of chondrocytes rather than acceleration of apoptosis in this layer.

We assessed whether Sirt6 deficiency would trigger chondrocyte senescence. The expression of intercellular-adhesion molecule-1 (ICAM-1) and plasminogen-activator inhibitor-1 (PAI-1) mRNA, the markers for cell senescence^25^, were evaluated by real-time RT-PCR analysis. As shown in figure 3k, Sirt6-depleted primary chondrocytes demonstrated higher levels of these genes. This result was consistent with Sirt6-silenced primary chondrocytes (supplementary figure S3a) and ATDC5 cells (supplementary figure S3b). Senescent cells secrete inflammatory cytokines, such as IL-1 and IL-6, as a result of a behavior termed the senescence-associated secretory phenotype (SASP)^26^. As expected, the expression of these cytokines was enhanced with Sirt6 deficiency (figure 3k and supplementary figure S3).

**Figure 4:**
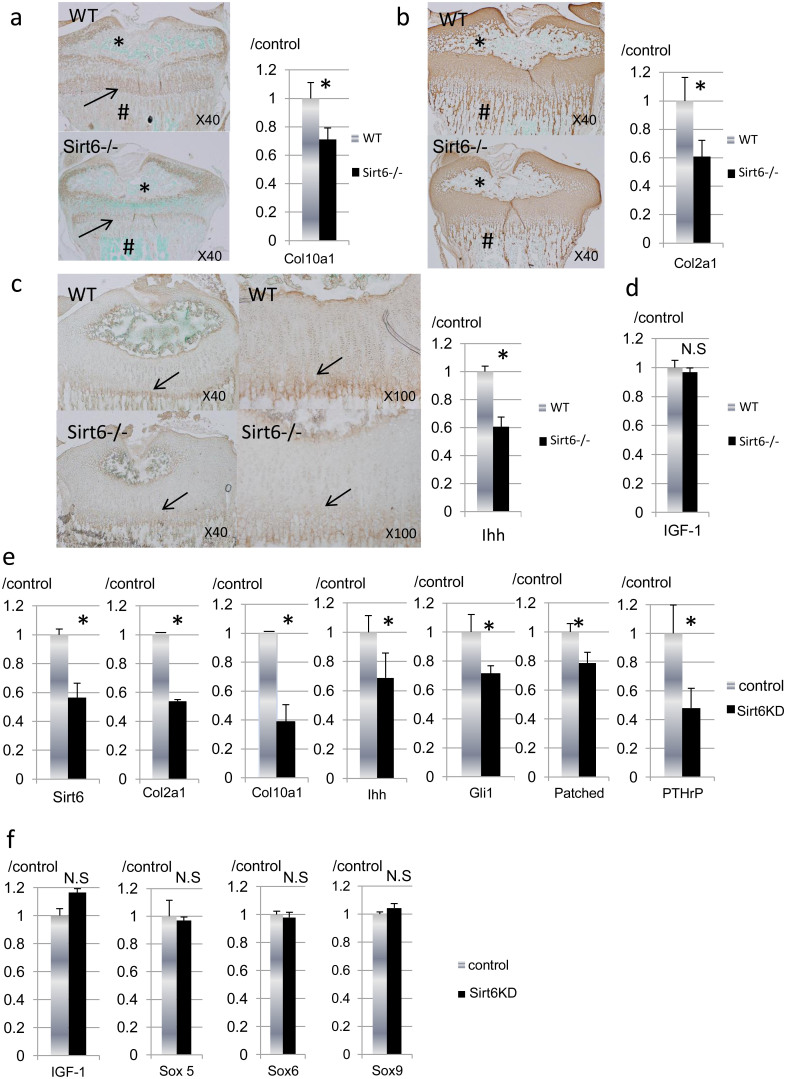
Effects of Sirt6 deficiency on chondrocyte differentiation. (a–c) Immunohistological findings and real-time RT-PCR analysis for type X collagen, type II collagen and Ihh. Data are normalized to expression levels in WT cartilage and β-actin (n = 4). Note markedly reduced expression of type X collagen and type II collagen in the secondary ossification center (a, b, *) and primary spongiosa (a, b, #). Ihh expression was decreased at the prehyper-hypertrophic zone (arrows) in Sirt6−/− mice. mRNA expression of Col10a1 (a), Col2a1 (b) and Ihh (c) was significantly reduced in primary epiphyseal chondrocytes from Sirt6−/− mice. (d) mRNA expression of IGF-1 in primary epiphyseal chondrocytes was comparable between WT and Sirt6−/− mice. (e), (f) Primary epiphyseal chondrocytes were treated with Sirt6 siRNA. Total RNA was isolated from the cells and used in real-time RT-PCR with the indicated primers. When Sirt6 was knocked down by siRNA in primary chondrocytes, expression of Col2a1, Col10a1, Ihh, PTHrP, Gli1 and Patched were all decreased (e). (f) The expression of Sox5, Sox6 and Sox9 and IGF-1 was not affected by Sirt6 knockdown. The graph shows relative levels of gene expression. Values represent the mean ± SD of 3 samples per group. *; p < 0.05. N.S; no significant. Magnification is indicated in the figure.

We next compared extracellular matrix (ECM) expression in growth plate cartilage and epiphyses by immunohistochemistry and real-time RT-PCR analysis (Figure 4). Staining for type X collagen, a marker for hypertrophic chondrocytes, was diminished in Sirt6−/− mice at the growth plate cartilage, primary spongiosa and secondary ossification center (Figure 4a). Similarly, the expression of type II collagen was markedly decreased in Sirt6−/− mice at the primary spongiosa and secondary ossification center (Figure 4b). Consistently, mRNA expression of Col10a1 (Figure 4a) and Col2a1 (Figure 4b) in epiphyseal chondrocytes was significantly reduced by Sirt6 deficiency. These data suggested that Sirt6 deficiency results in a delay of chondrocyte differentiation.

### Sirt6 agonizes Ihh expression in chondrocytes

Ihh is a key promoter of chondrocyte proliferation and differentiation^18^. Therefore, we assayed the expression of Ihh protein at the growth plate. Immunohistological analysis revealed that the expression of Ihh protein was weak in the Sirt6−/− growth plate (Figure 4c). Ihh mRNA expression was also reduced in Sirt6−/− primary chondrocytes (Figure 4c).

Sirt6 regulates hepatic synthesis of IGF-1, which plays important roles in chondrocyte differentiation and proliferation^27^,^28^,^29^,^30^. Thus, we evaluated endogenous IGF-1 expression in Sirt6−/− primary chondrocytes. As shown in figure 4d, IGF-1 expression was similar between chondrocytes derived from WT and Sirt6 null mice.

To avoid the effect of systemic factors on chondrocyte metabolism, we next examined the effects of acute Sirt6 gene knockdown on gene expression in cultured chondrocytes. When Sirt6 was knocked down by siRNA in primary epiphyseal chondrocytes, Ihh and its downstream targets (PTHrp, Gli1, Patched, Col2a1 and Col10a1) were all reduced (Figure 4e). In contrast, the expression of Sox5, Sox6 and Sox9, the other master genes for chondrocyte differentiation, was not affected by Sirt6 deficiency (Figure 4f). Furthermore, endogenous IGF-1 expression was not affected by Sirt6 silencing in vitro (Figure 4f).

### Impaired chondrocyte differentiation was rescued by restoration of Hh signaling in Sirt6 knocked down ATDC5 cells

**Figure 5:**
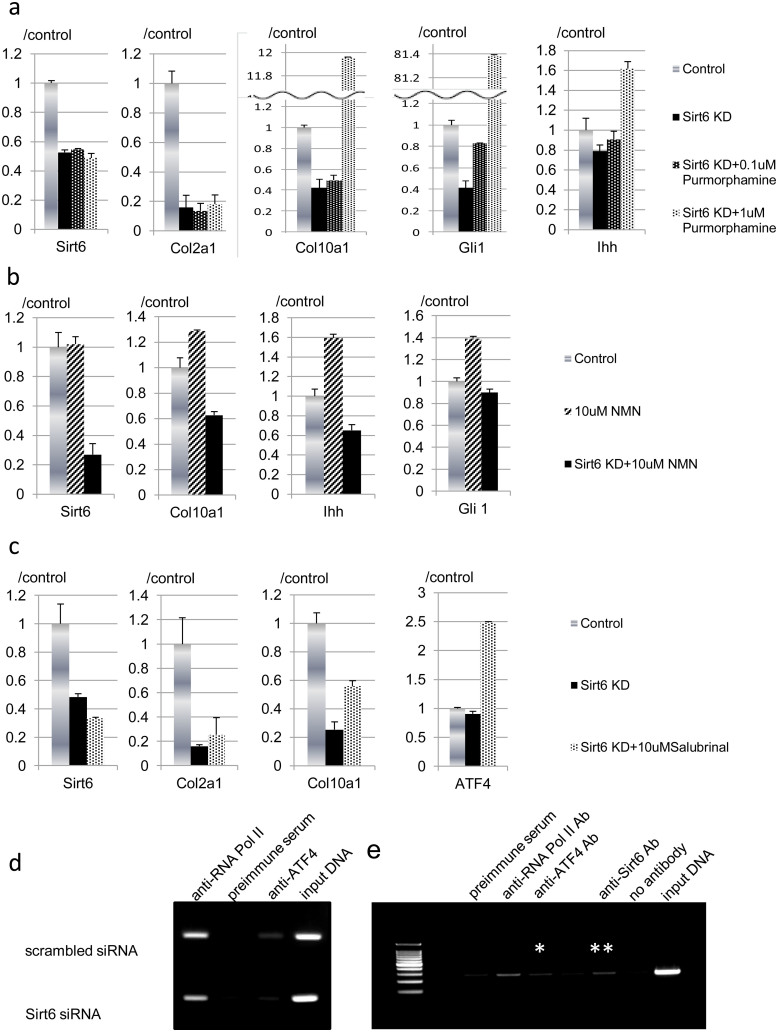
(a) Sirt6 promotes DNA binding of ATF4 to the Ihh gene promoter. (a), (b) ATDC5 cells were treated with Sirt6 siRNA and grown in differentiation medium for 7 days. Total RNA was isolated from the cells and used in real-time RT-PCR with the indicated primers. Ihh, Col2a1, Col10a1 and Gli1 were decreased by Sirt6 knockdown. Reduced expression of Col10a1, but not Col2a1, was rescued by purmorphamine (a). (b) NMN treatment enhanced Col10a1, Ihh and Gli1. These effects were clearly abolished by Sirt6 silencing. (c) Impaired expression of Col10a1, but not Col2a1, induced by Sirt6 silencing was partially restored by the administration of salubrinal with regard to restoration of Gli1 expression. ATF4 expression was enhanced by salubrinal treatment. (d) Control and Sirt6-silenced primary chondrocytes were processed for ChIP analysis using antibodies for ATF4. PCR was then performed with primers flanking the promoter for Ihh. Full-length blots are presented in Supplementary Figure S4. (e) Representative ChIP of *Ihh* with anti-ATF-4 Ab (*) and anti-Sirt6 Ab (**) and qRT-PCR of ChIP-ed in primary chondrocytes. The gels have been run under the same experimental conditions. The displayed data are representative of three experiments.

We further examined the action of Sirt6 on chondrocyte biology using chondrocyte-like ATDC5 cells to determine the status of Hh signaling activity by Sirt6 silencing. When Sirt6 was knocked down by siRNA in ATDC5 cells prior to inducing chondrocyte differentiation, Ihh and its downstream targets Col2a1, Col10a1 and Gli1 were decreased as seen in the experiments using primary chondrocytes (Figure 5a). Impaired synthesis of Col10a1 by Sirt6 knockdown was effectively reversed by purmorphamine, a synthetic compound that directly targets smoothened to activate Hh signaling^31^, in a dose dependent manner. These data indicate reactivation of Ihh signaling improves chondrocyte differentiation of Sirt6-depleted ATDC5 cells and confirm the ability of Sirt6 to enhance Ihh signaling. Whereas suppressed Col2a1 expression was not rescued by purmorphamine, Hh signaling reactivation upon purmorphamine treatment was confirmed by increased Gli1 expression in purmorphamine-treated ATDC5 cells as compared with vehicle-treated samples (Figure 5a).

Next, to study the effect of gain of Sirt6 activity on chondrocyte differentiation, nicotinamide mononucleotide (NMN), a key NAD+ intermediate, was administered to ATDC5 cells. NMN facilitates activity of sirtuins through enhancement of NAD biosynthesis^2^. NMN administration enhanced Col10a1 expression together with increased expression of Ihh and Gli1, and these effects were clearly abrogated by Sirt6 silencing (Figure 5b). These observations confirmed that Sirt6 regulates chondrocyte hypertrophy via upregulation of Ihh signaling.

### Sirt6 enhances DNA binding of ATF4 to the Ihh promoter

The transcription factor ATF4 regulates chondrocyte proliferation and differentiation via upregulation of Ihh expression in growth plate chondrocytes^24^. Thus, we focused on the role of Sirt6 in ATF4-mediated Ihh transcription. Downregulated Ihh signaling induced by Sirt6 knockdown was ameliorated by salubrinal, an ATF4 activator (Figure 5c). Impaired expression of Col10a1, but not Col2a1, was partially reversed by the administration of salubrinal (Figure 5c). These data also suggest that excess ATF4 expression in Sirt6-silenced ATDC5 cells was not adequate for the recovery of the differentiation defect of Sirt6-depleted ATDC5 cells.

The ChIP assay was conducted in the Ihh gene promoter to determine Sirt6 function in the modification of ATF4 binding to the Ihh gene promoter. Under basal conditions, the ATF4 protein was detected in the Ihh promoter DNA. When the Sirt6 signal was reduced by administering Sirt6 siRNA, ATF4 binding to the Ihh promoter was markedly decreased (Figure 5d). Furthermore, ChIP assay revealed Sirt6 protein bound to the Ihh promoter in the primary chondrocytes (Figure 5e). These data suggest that Sirt6 promotes DNA binding of ATF4 to the promoter region of the Ihh gene. These results indicate Sirt6 is positioned in the ATF4-Ihh axis for the regulation of chondrogenesis.

## Discussion

Sirt6−/− mice exhibit skeletal abnormalities, such as growth retardation, osteopenia and lordokyphosis. However, no detailed histological analyses on these mice have been previously performed. Thus, we analyzed the growth plate of Sirt6 −/− mice immunohistologically to investigate the actions of Sirt6 in chondrogenesis during postnatal skeletal development. Our data confirm a critical role for Sirt6 in sustaining the proliferation and differentiation of the chondrocyte and its interaction with the Ihh signaling pathway. Because the Sirt6–null phenotype in the growth plate partly resembles the phenotypes of inactive Ihh^20^, we investigated if Sirt6 could regulate the expression of the Ihh gene. Our in vivo and in vitro experiments indicate that Ihh is a downstream target of Sirt6 in chondrogenesis.

Immunohistological analysis indicated Sirt6 deficiency impaired the expression of PCNA in vivo. Furthermore, Sirt6 deficiency reduced the expression of cyclin D1 and cyclin D2 in chondrocytes in vitro. Cyclin D1 promotes chondrocyte proliferation, and cyclin D1-deficient mice exhibit a thin proliferative zone^32^, as also observed in Sirt6−/− mice. Sirt6−/− mouse embryonic fibroblastcultures and ES cell cultures exhibit a reduced proliferative rate^4^. Sirt6 acts by promoting DNA end resection, a critical step in double-strand break repair^7^,^33^. These previous data indicate that Sirt6 may regulate chondrocyte proliferation directly. On the other hand, cyclin D1 and cyclin D2 are also reported to be upregulated by Hedgehog signaling^34^. Ihh directly induces the proliferation of prehypertrophic chondrocytes^20^. Thus, Ihh may also be implicated in the down-regulated proliferation of chondrocytes in Sirt6−/− mice.

A prior study reports misexpression of Ihh under the control of collagen type 2 promoter impairs chondrocyte proliferation and delays Col10a1 mRNA expression^35^. Furthermore, several mutations that display aberrant expression of Ihh exhibit similar phenotypes, such as impaired chondrocyte maturation and proliferation^21^,^24^. These observations are very similar to our findings in Sirt6−/− mice, which suggests a link between Sirt6 and Ihh signaling.

Serum IGF-1 levels were markedly reduced in Sirt6−/−, even in 12-day-old Sirt6−/− mice^4^. Serum glucose, although normal in 12-day-old animals, decreased sharply afterwards and by day 24 reached the limit of detection^4^. As IGF -1 positively regulates Ihh expression in vivo^29^, we should rule out the effect of abnormal glycolysis or IGF signaling in Sirt6 null chondrocytes. To confirm whether Sirt6 directly regulates chondrocyte differentiation, we examined the effects of acute Sirt6 gene knockdown on gene expression in cultured primary chondrocytes and ATDC5 cells, under conditions where insulin was adequately supplied. As shown in Sirt6−/− mice-derived chondrocytes, the expression of the differentiation marker genes Col2a1 and Col10a1 was decreased in parallel with reduced Ihh expression by Sirt6 depletion, whereas endogeneous IGF-1 expression was not affected. Furthermore, aberrant expression of Col10a1 was effectively reversed by purmorphamine treatment, the agonist of smoothened^36^. These observations indicate that Sirt6 enhances Ihh expression in chondrocytes, and regulates chondrocyte hypertrophy via Ihh signaling. On the other hand, impaired Col2a1 could not be ameliorated by reactivation of Hh signal, indicating there may be other mechanisms independent of Ihh in the regulation of Col2a1 expression through Sirt6.

ATF4 directly regulates chondrocyte proliferation by affecting the transcription of Ihh^24^. ATF4 transactivates Ihh in chondrocytes, and ATF4 overexpression enhances endogenous Ihh mRNA synthesis^24^. Thus, we hypothesized that Sirt6 regulates Ihh secretion through ATF4 action. Salubrinal, a pharmacological inhibitor of eIF2α dephosphorylation, was employed to stimulate ATF4 mRNA synthesis in this study. A previous paper reports that in response to 10 μM salubrinal, an increase in mRNA levels of ATF4 with regard to elevation of the phosphorylation level of eIF2α is observed in MC3T3 osteoblastic cells^37^. Here we demonstrated that salubrinal partially rescued the expression of Col10a1 in Sirt6-silenced ATDC5 cells, however, excess ATF4 expression was not adequate for the full recovery of the differentiation defect of Sirt6-depleted ATDC5 cells. In combination with the results of the ChIP assay, we confirmed that Sirt6 enhances Ihh expression via ATF4 activation. However, it remains unknown how Sirt6 influences ATF4 binding to the promoter region of Ihh gene and whether it does so directly or indirectly. We failed to detect endogenous association of ATF4 and Sirt6 in primary chondrocytes by immunoprecipitation (data not shown). However, on the basis of existing information, it is tempting to speculate that Sirt6 may promote binding of ATF4 to the Ihh promoter by creating accessibility for cofactors via modification, deacetylation or ADP-ribosylation.

Sirt6 deficiency induces a cell-autonomous increase in glucose uptake and increases glycolysis^9^. Sirt6 functions as a corepressor of the transcription factor Hif1α and suppresses the expression of glycolytic genes, including GLUTs^9^. Chondrocytes are highly glycolytic cells and require an adequate supply of glucose for cell homeostasis^38^,^39^. Supporting these views, if increased glycolysis were the dominant factor affecting metabolism in Sirt6-null chondrocytes, chondrocyte differentiation should be facilitated in vitro, which is in contrast to our observations. These observations indicate that aberrant differentiation in Sirt6-deficient chondrocytes may be independent of regulation through HIF1α.

As neural-specific deletion of Sirt6 in mice shows postnatal growth retardation^10^, the implication of aberrant IGF-1 secretion in Sirt6 null mice should be counted in delayed growth. However, neural-specific Sirt6-deficient mice appear normal with body weight being comparable to that of littermates at birth^10^, whereas Sirt6 null mice exhibit dwarfism as shown here. Apoptotic chondrocytes are increased in the resting zone and in the proliferative zone of the long bones in IGF-1−/− mice^29^. In the growth plates of long bones, no difference in type II collagen and type X collagen expression between the IGF-I^−/−^ and IGF-I^+/+^ embryos was observed^29^,^28^. These observations are inconsistent with the phenotype of Sirt6−/− mice. These observations also indicated that local Sirt6 also contributes to skeletal growth via the regulation of chondrocyte metabolism in parallel with a systemic effect through IGF-1 secretion.

We also demonstrated Sirt6 protected chondrocytes from senescence. This finding was consistent with studies in endothelial cells^40^, induced pluripotent stem cells^41^, and fibroblasts^42^. Impaired proliferation and differentiation in Sirt6 null mice may be the result of senescence of chondrocytes at least in a part, Molecular mechanisms regulating cell senescence in chondrocyte are still to be elucidated.

Taken together, we found that Sirt6 has roles in the regulation of the proliferation and differentiation of chondrocytes. Currently, it is unknown if Sirt6 dominantly acts on chondrocyte metabolism through a direct effect in vivo. Furthermore, a direct effect of Sirt6 on chondrocytes in aged mice still remains to be elucidated. Future studies using chondrocyte-specific Sirt6 mutant mice will shed light on these questions.

## Methods

### Animals

Sirt6+/− mice were obtained from Jackson Laboratory (Bar Harbor, ME, USA). All animal experiments were approved by the Animal Care and Use Committee of Tokyo Medical and Dental University.

### Growth plate histology and histomorphometry

For paraffin embedded samples, hind limbs from 2-week-old Sirt6 −/− and littermate mice were dissected and processed for paraffin and methylmethacrylate embedding with and without decalcification, respectively, as previously described (Wu et al., 2009). Paraffin sections were stained with safranin O and hematoxylin and eosin (HE). Growth plate histomorphometry was performed on HE-stained sections of proximal tibia growth plates using Osteomeasure software (Osteometrics–Decatur). A representative field at 100 × was used to obtain cell counts, volume occupied by extracellular matrix and number of cells in the hypertrophic zone^43^, with slight modifications.

For immunofluorescent staining, each sample was embedded in 5% carboxymethyl cellulose (CMC) gel and completely frozen. Cryosections (5 μm) were produced on a Leica CM3050S Cryostat (Leica Inc, Germany) using a Cryofilm type IIC tape system (FINETEC, Japan).

### Cell culture conditions

The mouse chondrogenic ATDC5 cell line was obtained from the RIKEN cell bank (Tsukuba, Japan). Cells were maintained in DMEM/F12 (1:1) medium containing 5% fetal bovine serum (FBS), 10 μg/mL human transferrin (Invitrogen A/S, Tastrup, Denmark), and 3 × 10^−8^ M sodium selenite (Sigma-Aldrich, Copenhagen, Denmark) at 37°C in a humidified atmosphere containing 5% CO_2_. Chondrogenic differentiation of ATDC5 cells was induced as previously described^44^. Briefly, ATDC5 cells were seeded at a density of 6 × 10^3^ cells/cm^2^ in 6-well or 24-well plates and grown for 4 days. When cells became confluent, the medium was replaced with fresh medium supplemented with insulin (10 μg/mL).

Primary epiphyseal chondrocytes were isolated from 5-day-old mice as previously reported^45^. Briefly, cartilage tissues, including the femoral heads, femoral condyles and tibial plateau, were cut into small pieces and digested twice for 45 min each with 3 mg/ml type I collagenase. The cartilage pieces were then incubated in 0.5 mg/ml type I collagenase at 37°C in a thermal incubator with 5% CO_2_ overnight. The next day, cell aggregates were dispersed by pipetting. The cells were cultured in 12-well plates seeded with 5 × 10^4^ cells per well in DMEM/F12 medium containing 10% FBS and antibiotics.

ATDC5 cells were transferred to 6-well plates and, when 70% confluent, were transfected with 50 nM Sirt6 siRNA or scrambled siRNA with HiPerFect (Qiagen, Valencia, CA, USA) in medium supplemented with 10% FBS for 24 h. The target sequence of Sirt6 siRNA was 5'GAAGCUCCCAAUGCAAUAAAU3'(forward) and 5'UUAUUGCAUUGGGAGCUUCUG3' (reverse).

### RNA extraction and real-time RT-PCR

Total RNA was extracted from chondrocytes and cell lines using TRIzol (Invitrogen) according to the manufacturer's directions. Real-time PCR was performed using the SuperScript III Platinum Two-Step qRT-PCR kit with SYBR Green in the Mx3000P® QPCR System. Briefly, 0.5 μg total RNA was mixed with 10 μl 2× RT reaction mix and 2 μl RT, and then incubated for 50 min at 42°C. The reaction was terminated by heating for 5 min at 85°C. The cDNA mixture was then incubated for 30 min at 37°C in the presence of RNase H. PCR was carried out using a mixture of Platinum SYBR Green real- time RT-PCR Super-Mix UDG, template cDNA, 10 mM primers, and DNase-free H_2_O with a total volume of 10–20 μl per well. The cycling conditions were performed as indicated in the Invitrogen SuperScript™ III Platinum two-step real-time RT-PCR kit with SYBR Green. Gene expression was normalized to the endogenous control β−actin, and fold changes in the genes of interest were determined using the comparative threshold cycle (CT) method^46^.

### Immunohistochemistry

The protein expression of Sirt6, type X collagen (ColX), type II collagen (ColII), Ihh, activated caspase-3 and PCNA was determined by immunohistochemistry with anti-Sirt6 (1:50,) anti-ColX antibody (1:400, LSL, Japan), anti-ColII antibody (1:1000, Abcam Biochemicals, Cambridge, UK), anti-Ihh antibody (1:50, Santa Cruz Biotechnology, Santa Cruz, CA, USA), anti-activated caspase-3 (1:800, Cell Signaling Technology, USA) and anti-PCNA antibody (1:200, Abcam Biochemicals, Cambridge, UK), respectively, according to the manufacturer's instructions. Briefly, tissue sections were incubated overnight at 4°C with primary antibodies, followed by a 30-min incubation at room temperature with an appropriate biotinylated secondary antibody. Next, the signal was visualized using peroxidase-conjugated avidin and diaminobenzidine from a Vectastain kit, according to the manufacturer's instructions (Vector Laboratories, Burlingame, CA, USA). Sirt6 protein was detected by immunofluorescent staining using cryosections. Sections were incubated at 4°C overnight with anti Sirt6 primary antibody (1:50, Abcam Biochemicals, Cambridge, UK), followed by incubation with Alexa Fluor 594-labeled secondary antibody (Life Technologies, Inc) at 4°C overnight.

### Establishment of Sirt6-overexpressing chondrocytes

ATDC5 chondrocytic cells at 90% confluence were transfected with 150 ng Sirt6 or Ihh expression plasmid (pCMV-SPORT6.1-Sirt6, Invitrogen; or pCMV6-AC-GFP-Ihh, Origene) using Lipofectamine (Invitrogen). Twenty-four hours after transfection, cells were trypsinized and replated in DMEM/F12 (1:1) medium containing 5% FCS, 10 μg/ml human transferrin (Invitrogen A/S, Tastrup, Denmark), and 3 × 10^−8^ M sodium selenite (Sigma-Aldrich, Copenhagen, Denmark) and insulin (10 μg/ml) at 37°C in a humidified atmosphere containing 5% CO_2_ and cultured for three days before the analysis.

### TUNEL assay

Apoptotic cells in the growth plate of WT and Sirt6^−/−^ tibiae were detected by a TUNEL detection kit according to the manufacturer's instructions (Takara Shuzo, Kyoto, Japan). Briefly, tibia sections were incubated with 15 μg/ml proteinase K for 15 min at room temperature and then washed with PBS. The sections were immersed in TdT Enzyme diluted with Labeling Safe Buffer (provided in the kit) and then incubated for 90 min at 37°C in a humid atmosphere. After washing in PBS, the slides were examined by fluorescence microscopy.

### ChIP assay

Chromatin immunoprecipitation (ChIP) assay was performed using an EpiQuik Chromatin Immunoprecipitation (ChIP) kit according to the manufacturer's instructions (Epigentek Group Inc. NY. USA). Briefly, control and siRNA for Sirt6-treated primary chondrocytes were cross-linked with 1% formaldehyde in RPMI medium. The reaction was stopped with 1.25 M glycine. The cells were harvested and incubated in lysis buffer containing protease inhibitors, followed by DNA sonication. After centrifugation (14,000 × g for 10 min), an aliquot of the supernatant was incubated (2 h) with anti-ATF4 IgG or anti-Sirt6 IgG previously crosslinked to the 96-well strips. An aliquot of each supernatant was used as the input control. Positive and negative controls were processed using anti-RNA polymerase II IgG and anti-normal IgG, respectively. The immunocomplexes and input controls were incubated with proteinase K at 65°C. The samples were then transferred to the column and washed with 70 and 90% ethanol, and the purified DNA was eluted. To verify the binding of ATF4 or Sirt6 to the Ihh promoter^24^, PCR was carried out according to standard procedures. The primer sequences were as follows: 5_-GAGAAAGGGAATGTTGCCAG-3_ (forward1) and 5_-GTCTCTCCTTCCCGTTCCTT-3_ (reverse). The annealing temperature was fixed at 55°C.

### Statistical analysis

Data are expressed as the mean ± SD. Statistical analysis was performed with the Mann−Whitney U test or Bonferroni/Dunn test. p values < 0.05 were considered significant.

## Supplementary Material

Supplementary Information supplementary figures
