# Supplementary material for: Sirt6 regulates postnatal growth plate differentiation and proliferation via Ihh signaling
Source: Sci Rep. 2013 Oct 23;3:3022. doi: 10.1038/srep03022 (PMC6505680; doi:10.1038/srep03022)
Supplement: Supplementary Information — supplementary figures [file srep03022-s1.pdf]

Title; Sirt6 regulates postnatal growth plate differentiation and proliferation via Ihh signaling.

Jinying Piao[1], Kunikazu Tsuji[2], Hiroki Ochi[3], Munetaka Iwata[4], Daisuke Koga[5], Atsushi Okawa[5], Sadao Morita[1], Shu Takeda[6], Yoshinori Asou[5]

1; Department of Rehabilitation Medicine, Graduate School, Tokyo Medical and Dental University

1-5-45 Yushima Bunkyo-ku Tokyo Japan, 113-8519

2; Department of Joint Surgery and Sports Medicine

Section of Cartilage Regeneration, Tokyo Medical and Dental University, Tokyo, Japan

1-5-45 Yushima Bunkyo-ku Tokyo Japan, 113-8519

3; Laboratory of Veterinary Microbiology, Nippon Veterinary and Life Science University,

1-7-1 Sakaiminamicho Musashino-shi Tokyo Japan, 180-8602

4; Division of Veterinary Surgery, School of Veterinary Medicine, Nippon Veterinary and Life Science University

1-7-1 Sakaiminamicho Musashino-shi Tokyo Japan, 180-8602

5; Department of Orthopedics Surgery, Tokyo Medical and Dental University

1-5-45 Yushima Bunkyo-ku Tokyo Japan, 113-8519

6; Department of Physiology and Cell Biology, Tokyo Medical and Dental University

1-5-45 Yushima Bunkyo-ku Tokyo Japan, 113-8519

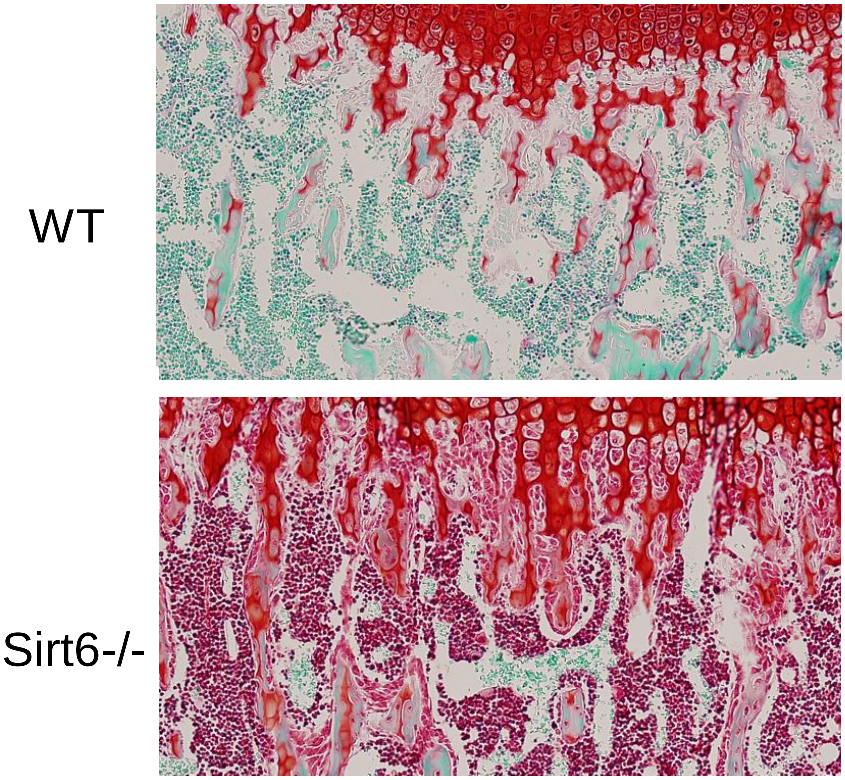

Supplementary Figure S1
Primary spongiosa from 3.5-week-old mice were stained with safranin O (X100).
Note the remarkable delay of ossification in Sirt6-/- mice.

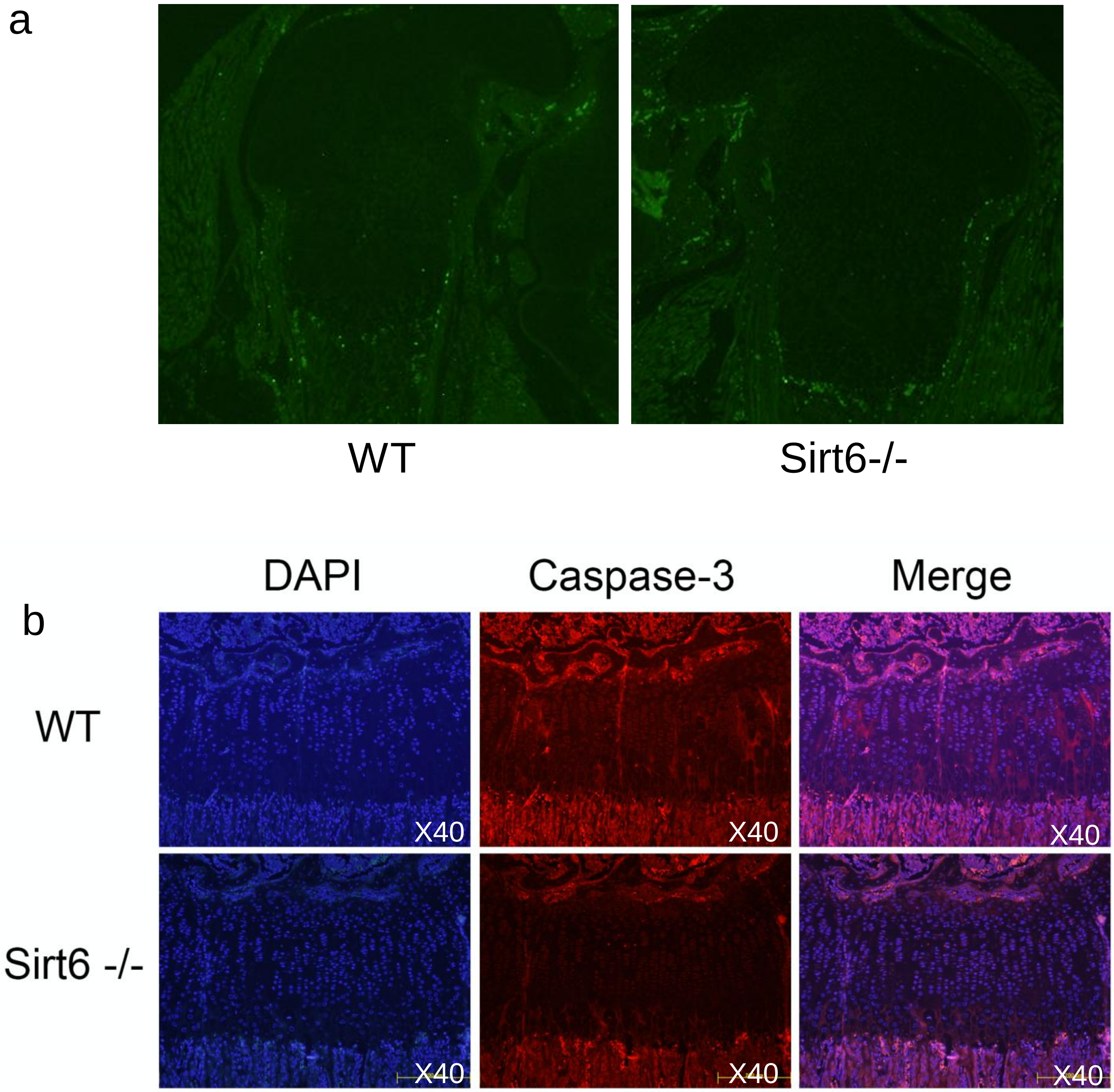

Supplementary Figure S2
(a)TUNEL staining of P1 WT and Sirt6-/- mouse femur sections.
The number of apoptotic cells was similar between Sirt6-/- and WT.
(b) Immunohistological findings for activated caspase-3 in P14.
Caspase-3 was not activated in the proliferating zone of both WT and Sirt6-/- mice.

Primary chondrocyte

a

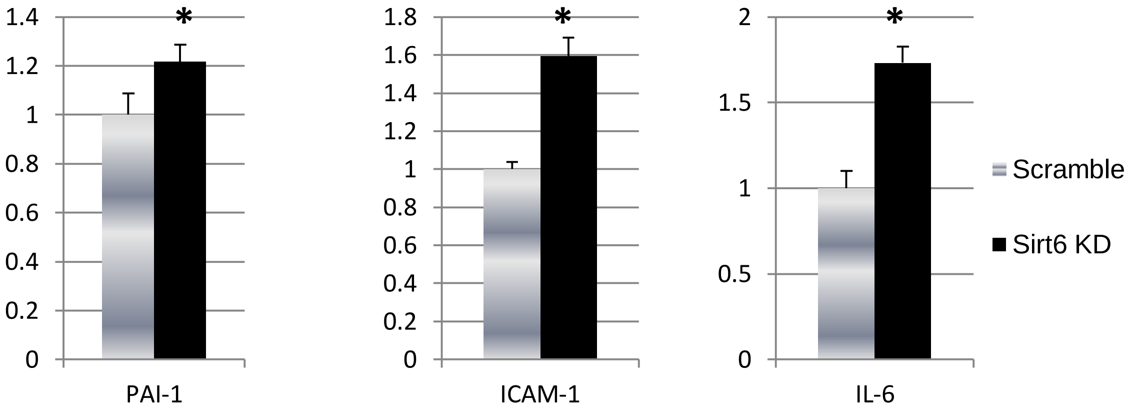

ATDC5 cells

b

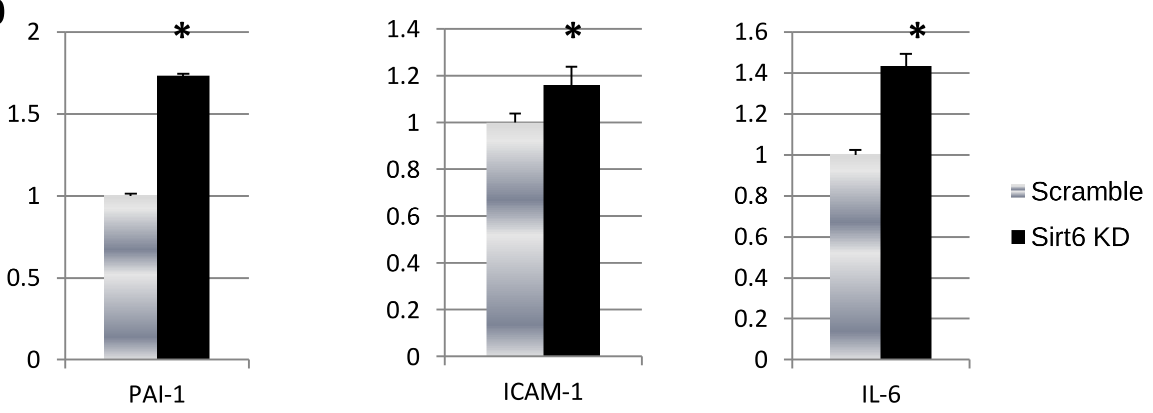

Supplementary Figure S3

mRNA expression of PAI-1, ICAM-1 and IL-6 was enhanced by Sirt6 knockdown in primary chondrocyte (a) and ATDC5 cells (b).

The graph shows relative levels of gene expression.

Values represent the mean ± SD of 3 samples per group. *; $p < 0.05$.

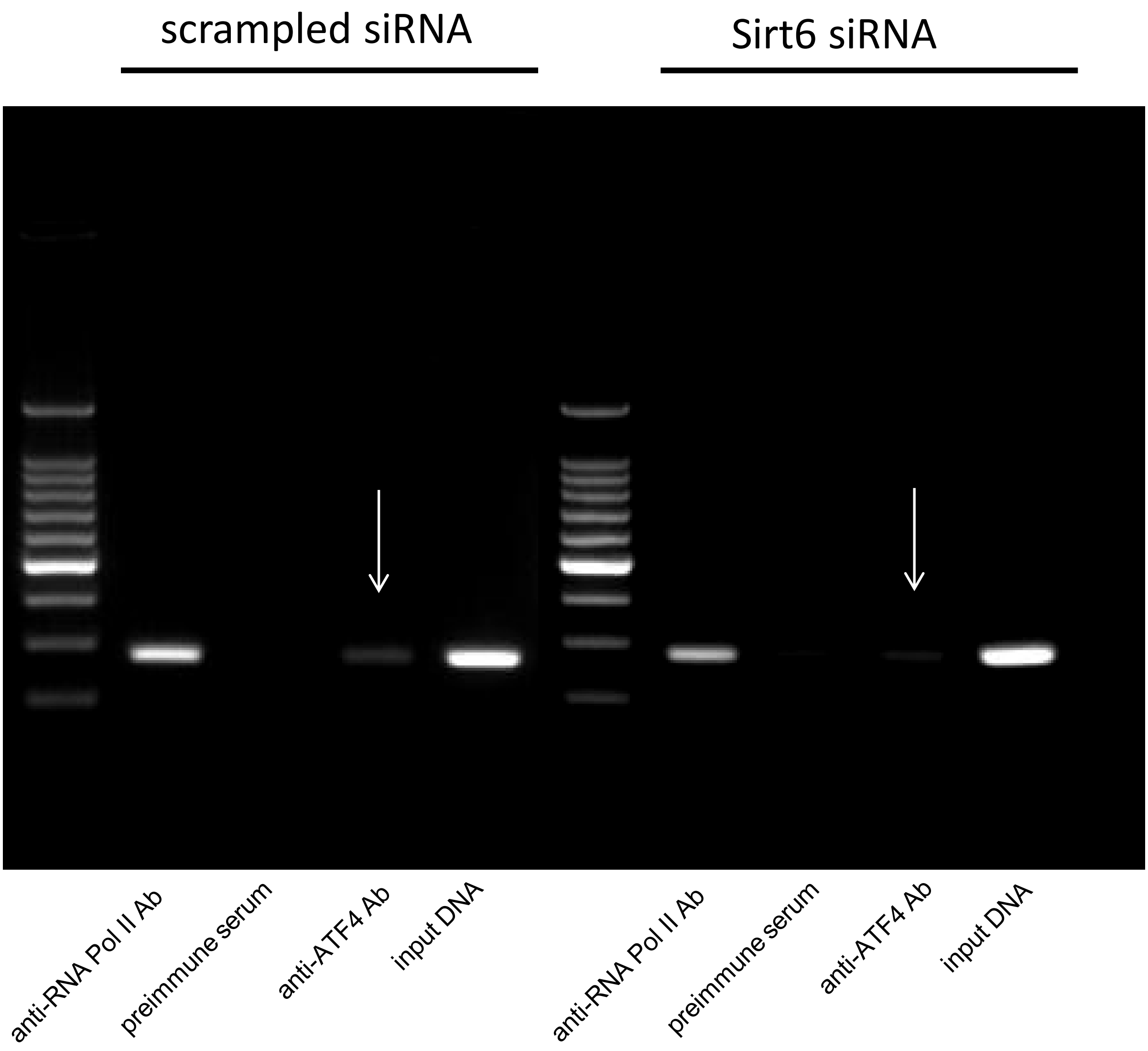

Supplementary Figure S4
Representative ChIP of *Ihh* with anti-ATF-4 antibody (Ab) and qRT-PCR of ChIP-ed in primary chondrocytes treated with scrampled siRNA (left panal) and Sirt6 siRNA (right panel).
